# Effectiveness of a digital educational system on the learners’ performance in preclinical fixed prosthodontic training

**DOI:** 10.1038/s41405-025-00344-6

**Published:** 2025-05-27

**Authors:** Lan-Anh Thi Pham, Tri Minh Doan, Thien-Thuy-Truc Tran, Van-Khoa Pham

**Affiliations:** 1 Department of Prosthodontics, Faculty of Dentistry, University of Medicine and Pharmacy at Ho Chi Minh City, Ho Chi Minh City, Vietnam; 2 Department of Operative Dentistry and Endodontics, Faculty of Dentistry, University of Medicine and Pharmacy at Ho Chi Minh City, Ho Chi Minh City, Vietnam

**Keywords:** Fixed prosthodontics, Dental foundation training

## Abstract

**Objectives:**

The purpose of this study was to evaluate the effectiveness of the digital education system on the crown preparation performance of dental students in repetitive preclinical training sessions, and students’ perceptions of the digital software in fixed prosthodontic practice.

**Material and methods:**

Forty dental students in the third year were recruited for the preclinical training of all-ceramic crown preparation on the lower right first molar in seven sessions. The first session (S1) included the initial didactic course and the first crown preparation practice with the instructor’s guidance. The second session (S2) comprised training in using the pedagogical Dental Teacher system. Students participated in five consecutive practice sessions (S2-S6), receiving only digital feedback. The seventh session (S7) was conducted with no digital or instructor support for all students. The abutment teeth were compared to the original tooth and measured via Exocad software. Each preparation received ten component scores corresponding to ten specific areas of the tooth and an overall score (out of 10) based on how well it matched the criteria. Participants were asked to complete a questionnaire survey to investigate their perception of the digital education software.

**Results:**

In the occlusal surface, the scores significantly differed in the third/fourth session compared to the first session (*p *= 0.037), and this difference remained throughout the study (*p *= 0.002 for the fourth session and *p* < 0.001 for the fifth, sixth, and seventh sessions). No significant difference was found for the buccal and lingual surfaces. In the proximal surfaces, the reduction scores of the occlusal second differed significantly between sessions 1 and 5 and between session 1 and session 6. However, no difference in scores from the last and first sessions was found. In the cervical second, no score difference was observed during the study. The overall scores of tooth removal rose from a baseline of 6.52 ± 0.79 to 7.14 ± 0.67 in session 5 (*p* = 0.033) and 7.35 ± 0.75 in session 6 (*p* < 0.001), before falling to 7.05 ± 0.74 in the last session (*p* = 0.203). Participants using digital software reported high satisfaction (92.5–95.0%) and expressed interest in future use for prosthodontic training (100%). However, over 50% of students assumed that digital software was incapable of substituting for teachers for guidance or assessment of crown preparation.

**Conclusion:**

The findings showed that the digital software improved students’ overall performance in preclinical prosthodontics and facilitated precision in some specific areas of tooth preparation. Preclinical crown preparation training benefits from the utilization of digital evaluation software; however, this digital pedagogic system cannot entirely replace the teachers’ roles.

## Introduction

Preclinical training in fixed prosthodontics is a fundamental part of the undergraduate dental curriculum. It focuses on developing students’ skills in preparing teeth for definitive restorations. Fixed prosthodontics education has prioritized comprehensive preparation skills because of the irreversible nature of tooth structure and the high demand for restorative care in daily practice [1]. Proper crown preparation involves minimizing tooth structure reduction to ensure sufficient thickness, strength, and contour of the restorative material while maintaining the integrity of the remaining tooth. Inadequate preparation of the dental structure can lead to mechanical failure and periodontal problems, thereby jeopardizing the long-term success of the dental crown/bridge [2]. Preclinical prosthodontics training is essential for dental students, building skills and confidence for clinical work with patients. Simulated practice enables students to enhance their fine motor skills, hand-eye coordination, and comprehension of tooth morphology within a controlled setting, unburdened by patient interaction. In conventional preclinical training programs, students use dental simulators and resin teeth to prepare the crown and subsequently, either the student or the instructor conducts a visual assessment of the reduction, convergence, margins, finishing lines, and preservation of adjacent teeth [3].

The preclinical dental curriculum has prioritized traditional teaching methodologies and criterion-referenced assessment [4]. Within the field of fixed prosthodontics, this method allows the instructors to provide constructive criticism of students’ tooth preparation, including their proficiency and the shortcomings requiring additional improvement [5]. Many dental schools have widely adopted this traditional training method for preclinical tooth preparation, with demonstrably effective outcomes [1, 6, 7]. However, this pedagogic approach lacks the objectivity to evaluate the extent of tooth structure removal required for optimal preparation [8–10]. The inconsistencies in how different faculty members grade the same student’s project reveal the inherent subjectivity of traditional assessment methods [8]. The process of students receiving instructor feedback is time-consuming, given the high student-to-instructor ratio. No real-time feedback during tooth preparation impedes students’ capacity to analyze their performance and enhance their practical proficiency [11]. The individual assessment format also leads to teacher fatigue from excessive speaking and student fatigue because of extended waiting periods [1].

Recently, the utilization of digital softwares, via scanners, virtual simulators, and computer-aided design and manufacturing (CAD-CAM) software has become increasingly prevalent. Digital applications are also integrated into the dental school curriculum, and several digital evaluation softwares have been applied in preclinical practical training [1, 9, 12, 13]. Current trends in digital dentistry suggest that technology may play a significant role in enhancing student learning outcomes [14]. With computer-aided systems, there are many opportunities for students to receive immediate feedback on their preparation designs and assess their manual skills, fostering a more effective and self-directed learning experience [1]. Digital assessment systems offer objective scoring, comparing student work to ideal examples and thereby improving self-assessment skills crucial for effective learning. Implementing digital softwares can lead to a reduction in faculty working hours and a significant positive effect on the dental curriculum [15, 16]. Existing literature suggests that digital assessment surpasses conventional assessment to improve students’ skills [9, 13, 17]. Three-dimensional digital softwares helped to improve students’ self-assessment skills, especially those of underperforming students [1, 18].

**Fig. 1:**
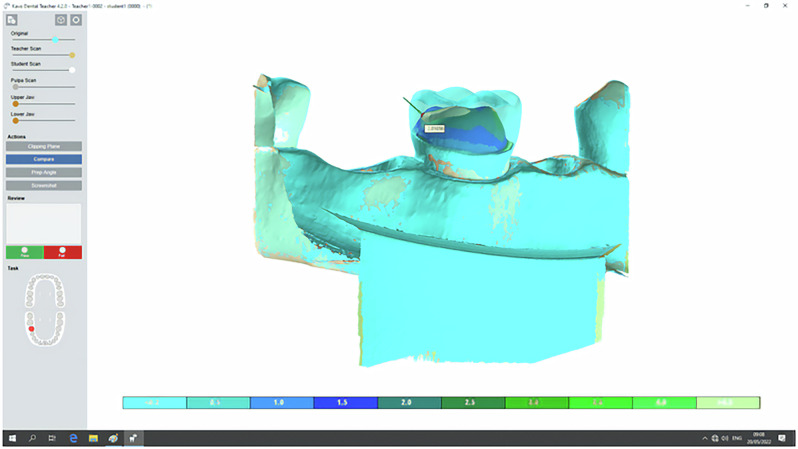
Tooth reduction visualization in Dental Teacher software. The color band under the tooth preparation shows the corresponding millimeters of reduction. Upon cursor placement on any position of the tooth preparation, the software displays the precise numerical reduction level.

Globally, digital surface mapping systems are among the most prevalent digital educational softwares in dental schools, demonstrating efficacy in preclinical student training. Following the process of model scanning, a three-dimensional topographical map is then displayed on the screen for the dental students to examine and learn from. This virtual image helps students to identify shortcomings in their preparations, refine their techniques, and enhance their future professional practices [19]. One of these softwares is, for example, the Dental Teacher software (KaVo, Biberach, Germany), which is used to compare the student’s preparation with the anatomical tooth or the master preparation of the teacher. This software provides fast, objectively measurable results displayed in clear graphics with high magnification (Fig. 1) [5, 20]. While some studies reported significant benefits, others demonstrated no clear advantage over traditional instructor-led feedback. The teacher’s opinions and advice continue to be of paramount importance in fostering the development of their students’ performance and self-assessment skills [1, 8, 19]. Moreover, studies often emphasized the evaluation of final outcomes in tooth preparation [1, 4, 8] while this process is a prolonged journey, requiring multiple repeated formative practice sessions to develop the necessary dexterity and proficiency [5, 21].

Despite increasing interest in simulation-based education, the literature remains limited in addressing how digital systems support autonomous learning throughout this prolonged learning curve. Therefore, this study aimed to evaluate the effectiveness of the digital educational system in improving students’ crown preparation performance during repetitive preclinical training sessions. The null hypothesis was that the digital software did not improve the performance scores of dental students in the preclinical fixed prosthodontic training. The study further examined students’ innovative learning experiences within digital pedagogical approaches.

## Materials and methods

### Subjects

The sample size was determined using a before-after (paired) design formula:

$$n\ge \frac{{(({Z}_{1-\alpha /2}+{Z}_{1-\beta })S\sqrt{2(1-{r}_{within})})}^{2}}{{E}^{2}}$$

Based on the study by Yu et al. (2022) [1], the mean pre-intervention score was 71.4 ± 6.2, and the mean post-intervention score was 76.9 ± 7.6, yielding a mean improvement of 5.5 points. Assuming a two-tailed significance level (α) of 0.05, a statistical power (1−β) of 0.8, an estimated value r = 0.5 and applying the sample size calculation formula, it was determined that a minimum of 13 students were required. During the recruitment process for study participants, we received registrations from 40 third- year students. Therefore, the study sample consisted of 40 students. In this third-year of a six-year curriculum, students completed the Preclinical Fixed Prosthodontics Crown module, gaining simulation experience but lacking clinical practice. The study was approved by the Ethics Committee for Biomedical Research, University of Medicine and Pharmacy at Ho Chi Minh City (No 947/HĐĐĐ-ĐHYD). Informed consent was obtained from all participants involved in this study. Prior to participation, all participants were provided with a detailed explanation of the study’s objectives, procedures, potential risks, and benefits. Participants were given sufficient time to consider their participation, ask any questions they had, and were informed of their right to withdraw from the study at any point without any consequence.

### Study design

In the first session (S1), the students were asked to take a didactic course, including the PowerPoint presentations of operational instructions and assessment criteria for making an all-ceramic crown. Students also completed the initial preparation on the lower right first molar within two hours with the instructor’s guidance. For the practical training, each participant received high-speed and low-speed handpieces, a set of diamond burs (Mani, Tochigi, Japan), a periodontal probe, putty indexes, and typodonts (Nissin Dental Products, Kyoto, Japan). Students only used the instruments provided to prepare the all-ceramic crown in a simulated phantom head within two hours of each session. The primary method of assessing tooth preparations involved the use of a putty index and a periodontal probe with marking intervals at 1 mm.

In the second session (S2), students were trained carefully to use Dental Teacher software (KaVo, Biberach, Germany) for the digital assessment. This automated software displayed the structure removal of all tooth surfaces when compared to an original tooth as reference data. This visualization used different colors to represent the corresponding reduction in millimeters (Fig. 1). Students participated in five consecutive practice sessions with digital software (2^nd^ session–6^th^ session). Following the operational manual, they completed the procedure step-by-step and autonomously assessed their preparations using the software’s feedback throughout each session.

**Fig. 2:**
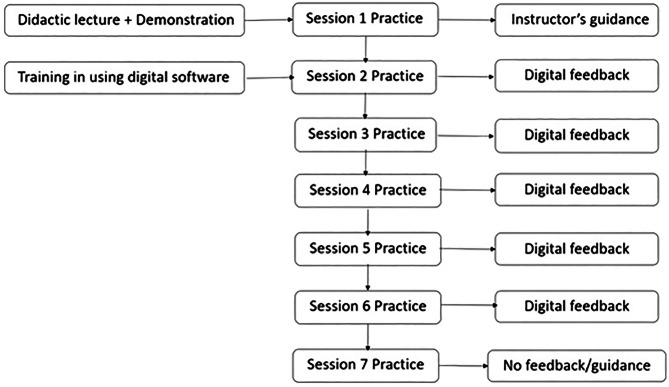
Flowchart of the study.

Finally, the 7^th^ session (S7) involved a summative crown preparation, conducted entirely without feedback from the instructor or the digital software. Students used the same rubric form to self-assess their preparations during all training sessions (Table 1). Rubric assessment form evaluated ten areas of five tooth surfaces (two areas per surface), using a 0–1 ranking scale with detailed descriptions of reduction for each area. Figure 2 presents the study’s flowchart.

**Table 1:** The assessment criteria of tooth reduction for the all-ceramic crown of the first lower molar.

Area of interest	Excellent	Clinically acceptable	Not clinically acceptable	Score
	(1 point)	(0.5 points)	(0 points)	
Occlusal surface				
Buccal second	1.0–1.5 mm	1.5–2.0 mm or	>2.0 mm or	1
		0.5–1.0 mm	<0.5 mm	
Lingual second	1.0–1.5 mm	1.5–2.0 mm or	>2.0 mm or	1
		0.5–1.0 mm	<0.5 mm	
Buccal surface				
Cervical second	0.8–1.2 mm	1.2–1.5 mm or	>1.5 mm or	1
		0.5–0.8 mm	<0.5 mm	
Occlusal second	1.0–1.5 mm	1.5–2.0 mm or	>2.0 mm or	1
		0.5–1.0 mm	<0.5 mm	
Lingual surface				
Cervical second	0.8–1.2 mm	1.2–1.5 mm or	>1.5 mm or	1
		0.5–0.8 mm	<0.5 mm	
Occlusal second	1.0–1.5 mm	1.5–2.0 mm or	>2.0 mm or	1
		0.5–1.0 mm	<0.5 mm	
Mesial surface				
Cervical second	0.8–1.2 mm	1.2–1.5 mm or	>1.5 mm or	1
		0.5–0.8 mm	<0.5 mm	
Occlusal second	1.3–1.7 mm	1.7–2.0 mm or	>2.0 mm or	1
		1.0–1.3 mm	<1.0 mm	
Distal surface				
Cervical second	0.8–1.2 mm	1.2–1.5 mm or	>1.5 mm or	1
		0.5–0.8 mm	<0.5 mm	
Occlusal second	1.3–1.7 mm	1.7–2.0 mm or	>2.0 mm or	1
		1.0–1.3 mm	<1.0 mm	
Total				10

#### Evaluation method of crown preparations

To minimize uncontrolled variables, the same trained researcher scanned all prepared teeth from all sessions using Arctica Autoscan (KaVo, Biberach, Germany). An independent staff member, uninvolved in the research, coded all tooth preparations and scanned files. Only one researcher measured all scanned files with Exocad software (Exocad GmbH, Germany).

**Fig. 3:**
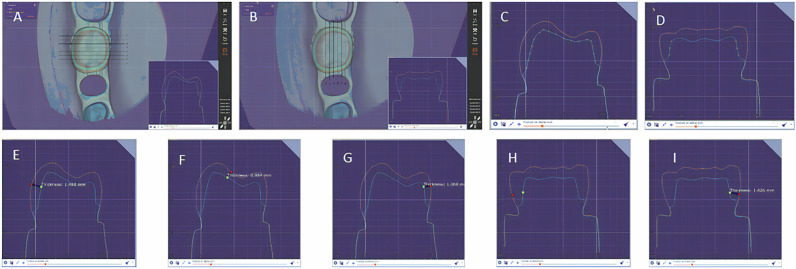
Digital measurement procedure with the Exocad software. **A** Eight bucco-lingual cross-sections were based on horizontal lines. **B** Six mesio-distal cross-sections were based on vertical lines. The image in the lower right corner of the screen is the cross-section of the corresponding slice. **C** Point of measurements for buccal, occlusal, and lingual reduction in each bucco-lingual cross-section. **D** Point of measurements for mesial and distal reduction in each mesio-distal cross-section. **E**–**I** Using the “Measurement tools: Thickness” function of Exocad to measure the tooth reduction of five surfaces: buccal surface, occlusal surface, lingual surface, mesial surface, distal surface.

An unprepared tooth was scanned as the index tooth for all measurements. The software Exocad enables the superposition of encoded scan data of the prepared tooth with this index tooth to assess the axial/occlusal removal. Subsequently, it was necessary to make eight buccolingual cross-sections and six mesiodistal ones at the specific lines for reproducibility. For each selected cross-section, the measurement tool “Thickness” of the software was used to quantify the tooth reduction at several points of agreement on the grid line (Fig. 3).

Each point’s score was thus determined by applying the assessment criteria with three score levels (0, 0.5, or 1) (Table 1). To produce component scores for each area of interest and an overall score, the researchers averaged the scores from these point analyzes. Ten areas of interest included the cervical second and the occlusal second for four axial surfaces; the buccal second and the lingual second for the occlusal surface. The evaluators assigned a total score between 0 and 10 after evaluating the crown preparation and grading ten areas.

### Questionnaire

Following the study, students answered a ten-question survey about their perceptions of the digital education system (Fig. 5A). The surveys comprised four sections; three questions on the efficacy of the digital software to tooth preparation performance (Q1-Q3), three questions on the efficacy of the digital software to assessment competency (Q4-Q6), two questions on the students’ interest in the digital educational software (Q7, Q8), and two last questions on teachers’ roles in the digital context (Q9, Q10). A five-point Likert scale was used to assess stated opinions. The responses ranged from “I strongly agree” to “I strongly disagree”.

### Statistical analysis

Statistical analysis was carried out using SPSS Statistics Version 26.0 (IBM Corp., Armonk, NY, USA) with a significance level of *p* < 0.05. The Kolmogorov–Smirnov Test was used to prove normality.

SPSS reliability analysis of intra-class correlation coefficient (ICC) (two-way mixed effects model, absolute agreement definition) determined the researcher’s consistency in measuring student tooth preparations by Exocad software.

A comparison of assessment results throughout seven practical sessions was conducted using Friedman tests with Bonferroni’s correction for multiple repeated comparisons (α = 0.05).

## Results

Forty third-year dental students (21.84 years old on average; 17 males, 23 females) participated in the research. The researcher who graded the scanned files showed good calibration, achieving ICC values of 0.87 (95% CI 0.86–0.88).

### Tooth preparation scores through seven practical sessions

The tooth removal data was compared to each area of interest criteria and calculated an overall score. Table 2 presents the component scores and the overall score from all seven consecutive training sessions.

**Table 2:** The scores of the preparation results in seven sessions and the comparison of the first session and six remaining sessions.

Area	Tooth reduction scores (Mean-SD)							*p* value* (overall)
	Session 1 (S1)	Session 2 (S2)	Session 3 (S3)	Session 4 (S4)	Session 5 (S5)	Session 6 (S6)	Session 7 (S7)	
**Occlusal surface**								
Buccal second	6.45 (1.27)	7.15 (1.30)	7.37 (1.07)	7.65 (1.00)	7.78 (1.09)	7.93 (0.81)	7.88 (0.93)	
*p value (S1-Sn)*	*–*	*0.478*	***0.037***	***0.002***	***<0.001***	***<0.001***	***<0.001***	***<0.001***
Lingual second	6.35 (1.90)	7.30 (1.72)	7.64 (1.02)	8.00 (0.96)	7.80 (1.04)	8.34 (0.90)	7.79 (1.00)	
*p value (S1-Sn)*	*–*	***0.016***	*0.056*	***<0.001***	***<0.001***	***<0.001***	***<0.001***	***<0.001***
**Buccal surface**								
Cervical second	5.97 (1.49)	5.81 (1.48)	5.82 (1.37)	5.71 (1.54)	6.01 (1.56)	6.28 (1.60)	5.86 (1.44)	
*p value (S1-Sn)*	*–*	*–*	*–*	*–*	*–*	*–*	*–*	*0.766*
Occlusal second	6.46 (2.13)	6.44 (2.15)	6.08 (1.88)	6.27 (2.45)	6.79 (1.86)	7.22 (1.92)	6.95 (1.75)	
*p value (S1-Sn)*	*–*	*1.000*	*1.000*	*1.000*	*1.000*	*0.219*	*1.000*	***0.012***
**Lingual surface**								
Cervical second	7.26 (1.38)	7.83 (1.46)	7.92 (1.00)	7.85 (0.92)	8.02 (1.16)	7.84 (1.28)	7.77 (1.00)	
*p value (S1-Sn)*	*–*	*–*	*–*	*–*	*–*	*–*	*–*	*0.143*
Occlusal second	7.88 (1.39)	8.05 (1.17)	8.34 (1.04)	8.41 (1.00)	8.43 (1.22)	8.37 (1.13)	8.08 (1.06)	
*p value (S1-Sn)*	*–*	*–*	*–*	*–*	*–*	*–*	*–*	*0.070*
**Mesial surface**								
Cervical second	6.53 (1.65)	6.33 (1.63)	6.56 (1.01)	6.70 (1.08)	6.81 (1.18)	6.84 (1.08)	6.36 (1.27)	
*p value (S1-Sn)*	*–*	*–*	*–*	*–*	*–*	*–*	*–*	*0.482*
Occlusal second	5.95 (1.51)	6.57 (1.31)	6.71 (1.02)	6.87 (0.95)	6.82 (0.99)	6.94 (0.99)	6.67 (1.07)	
*p value (S1-Sn)*	*–*	*0.914*	*0.447*	*0.138*	***0.047***	***0.019***	*1.000*	*0.022*
**Distal surface**								
Cervical second	6.43 (1.39)	6.05 (1.73)	6.41 (1.37)	6.44 (1.53)	6.49 (1.63)	6.66 (1.49)	6.41 (1.41)	
*p-value (S1-Sn)*	*–*	*–*	*–*	*–*	*–*	*–*	*–*	*0.410*
Occlusal second	5.94 (1.18)	6.37 (1.25)	6.44 (1.26)	6.67 (1.20)	6.43 (1.08)	7.06 (0.95)	6.72 (1.23)	
*p value (S1-Sn)*	*–*	*1.000*	*1.000*	*0.273*	***0.003***	***0.030***	*0.219*	***0.008***
**Overall**	6.52 (0.79)	6.79 (0.91)	6.93 (0.71)	7.06 (0.73)	7.14 (0.67)	7.35 (0.75)	7.05 (0.74)	
*p value (S1-Sn)*	*–*	*1.000*	*1.000*	*0.064*	***0.033***	***<0.001***	*0.203*	***<0.001***

*:Related-samples Friedman’s two-way analysis of variance by ranks, Bonferonni correction, comparing the score of session 1 to six remaining sessions.

The occlusal surface (OS) was divided into the buccal second (Bs) and the lingual second (Ls). In the buccal second, a statistically significant difference was observed in scores of the third session compared to the first session (*p *= 0.037), and this difference remained throughout the study: *p *= 0.002 for the fourth session and *p* < 0.001 for the fifth, sixth, and seventh sessions. The score increased from 6.45 ± 1.27 to 7.88 ± 0.93 at the end of the study. In the lingual second, the score increased from 6.35 ± 1.90 at the baseline to 8.00 ± 0.96 in the fourth session (*p* < 0.001) and this difference continued to the last session (7.79 ± 1.00; *p* < 0.001).

The researchers divided the axial surfaces into the occlusal second (Os) and the cervical second (Cs). In the buccal surface (BS), the reduction score of the cervical second is among the lowest scores (5.97 ± 1.49), persisting until the seventh session (5.86 ± 1.44, *p* = 0.766). The score of the occlusal second increased gradually to the sixth session, with a significant difference in tooth reduction scores among the seven sessions (*p* = 0.012). However, comparing session one with the remaining six sessions showed no difference. The component scores of the lingual surface (LS) were the highest at the baseline (7.26 ± 1.38 for CS and 7.88 ± 1.39 for OS), and the subsequent progressive change in the following practical training, but the inter-sessional differences were not significant (*p* = 0.143 for CS and *p* = 0.07 for OS).

In the proximal surfaces, the reduction scores of the occlusal second differed significantly between sessions 1 and 5 (*p* = 0.047 for the mesial surface-MS, *p* = 0.003 for the distal surface-DS). Session six had the highest scores of all seven sessions (6.94 ± 0.99 for MS and 7.06 ± 0.95 for DS); these scores were significantly higher than session one’s scores (*p* = 0.019 for MS, *p* = 0.030 for DS). However, no difference in scores from the last and first sessions was found. In the cervical second, no score difference was observed during the study.

**Fig. 4:**
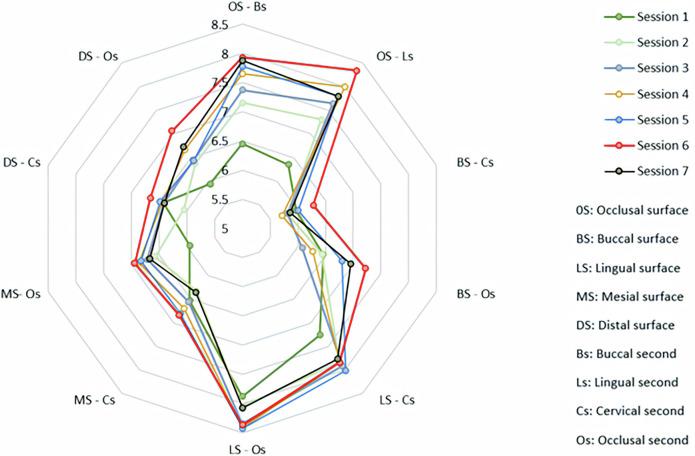
Variation of scores during seven training sessions: the tendency to rise continuously from the first session to a peak in the 6^th^ session, then decline in the 7^th^ session.

The overall scores of tooth removal rose from a baseline of 6.52 ± 0.79 to 7.14 ± 0.67 in session 5 (*p* = .033) and 7.35 ± 0.75 in session 6 (*p* < 0.001), before falling to 7.05 ± 0.74 in the last session (*p* = 0.203). Figure 4 illustrated the tendency of scores to rise continuously from the first session to a peak in the 6^th^ session, then decline in the 7^th^ session.

### Student opinions on the digital educational software for preclinical practice

**Fig. 5:**
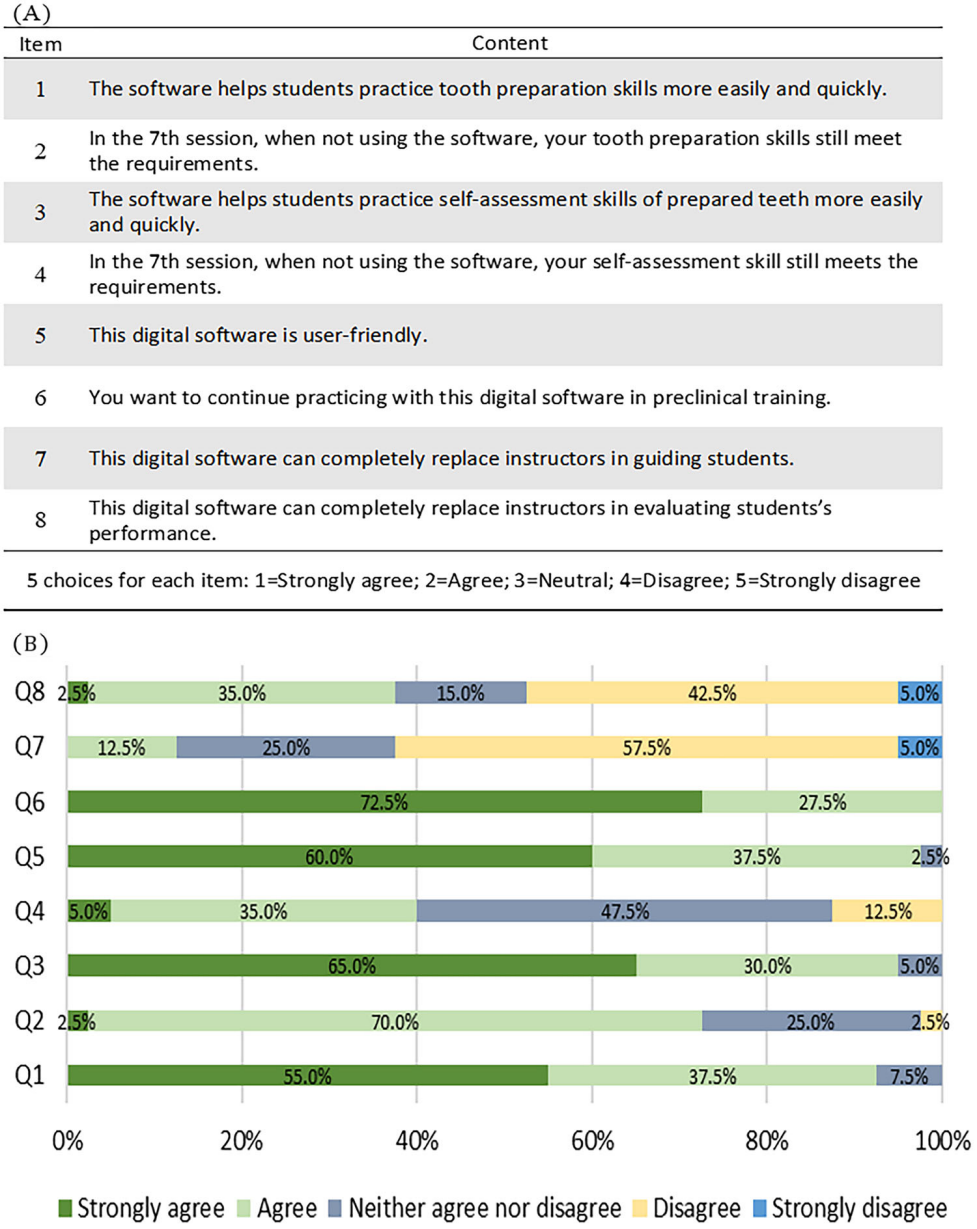
Questionnaire and students' perceptions of digital application in preclinical fixed prosthodontics. **A** The questionnaire items evaluating students' perceptions of digital applications in preclinical fixed prosthodontic practice. **B** The chart illustrating the distribution of students’ responses to each questionnaire item.

Forty participants responded to the questionnaire, and the results are shown in Fig. 5B. Most surveyed students (*n* = 37, 92.5%) either agreed or strongly agreed that the Dental Teacher software enhanced their performance and facilitated efficient tooth preparation. Nearly all students (95%, 38 of 40) reported that the digital software significantly aided their assessment of practical tasks conveniently and effectively. The digital software received strongly positive student feedback, with 97.5% (*n* = 39) of respondents emphasizing its ease of use. All students preferred the digital evaluation systems as a training software for tooth preparation and expressed the desire for continued practice using the digital softwares. However, in the summative test (no digital aids), 27.5% of students reported their insufficient tooth preparation skills and more than half of the students expressed uncertainty about assessment skills. When considering the role of instructors in digital training environments, 62.5% of students expressed the view that digital assessment cannot replace the instructor’s guidance in the preclinical laboratory setting. 47.5% of students believed digital softwares should not be the only assessment method.

## Discussion

### Effectiveness of the digital software in improving student’s performance of tooth preparation

Preclinical fixed prosthodontic training is essential for students to improve their skills in operational procedures and dexterity. Nowadays, with the development of computer-based technology, interactive digital dental softwares give students real-time visual feedback as they explore three-dimensional tooth preparation in the software’s interface [22]. This study aimed to determine the effectiveness of the Dental Teacher system in dental students’ repetitive preclinical training on all-ceramic crown preparation.

The study revealed that this digital software may significantly improve the overall outcome of tooth reduction for all ceramic crowns. Undergraduate students of the experimental group, who used merely the Dental Teacher system during five practical training sessions, achieved tooth preparation scores in session 5 and session 6 significantly different from those in the first session, validating the system’s preclinical training effectiveness after multiple repetitive sessions. Nagy et al. [23] investigated the impact of digital feedback using the Dental Teacher software on preparing onlays, compared to the conventional group. The experimental group demonstrated significant improvements in all parameters, while the control group showed similar scores between the first and second preparation attempts [23]. The other previous studies also showed that the Dental Teacher software enabled an efficient educational method for learning preclinical skills [24, 25]. The primary benefits were significant magnification, three-dimensional visibility, and the capacity for quantifying objective measurements in different planes and directions. The digital software allowed them to identify errors, make relevant corrections, and achieve higher scores [1, 14]. Students used the innovative pedagogical softwares as self-training aids, and had the freedom to set their own learning pace, as some studies suggested [12, 23, 26]. The digital software provided a simulated self-directed learning environment for interactive student exercises, unlike traditional textbooks or teacher-led instructions [14, 26, 27]. Nowadays, the rising student-to-faculty ratio necessitates students’ independent learning with digital software, compensating for the faculty shortage and potential bias in crown evaluations [28]. The benefits above show that digital software encourages tooth preparation skills as well as student-centered learning and strengthens students’ self-critical skills.

However, different training effects among tooth areas were observed in this study. Occlusal reduction showed improvement after the 3^rd^ training session (for the buccal second) and 4^th^ session (for the lingual second), maintaining until the end of the study. The assessment using the digital software and putty index showed clear vision in the buccal second, while the lingual second was less visible, delaying the improvement. The cervical second of the proximal surfaces is generally harder to prepare than the occlusal surface, considering the ease of access to manipulate and evaluate by visualization. This finding is also in line with previous research results, in which the students scored lowest on axial reduction [3, 29, 30]. The study by Rosella et al. highlighted that precise control of tooth tissue removal depth and direction posed a significant challenge for prosthodontists [31]. Therefore, to enhance students’ crown preparation performance, a 4-session digital training is sufficient for occlusal clearance, while longer practice will be beneficial for axial reduction.

Digital applications have an additional problem of time consumption. Implementing digital softwares in dental schools will necessitate a period of adjustment during which faculty train students on the software: adequate time and training are essential to optimizing the scan and assessment flow. Students of the digital training might need time to familiarize themselves with the novel digital evaluation system; consequently, the actual practical exercise time was reduced [16, 32]. Gratton et al. [33] revealed that even with a student-to-scanner ratio of 10:1 considered satisfactory, students did not have sufficient time to familiarize themselves with the new software [33]. In our research, the ratio was approximately 10:1. As the practical sessions progressed, students became more proficient in using the scanner and software, allowing them less time for assessment of their preparation and more time for refinement. Consequently, their scores showed a statistically significant improvement in session 5 and session 6.

Contrasting with the studies mentioned above, some researchers indicated digital softwares are insufficient to improve autonomous student performance in crown preparation [9, 17, 19, 33]. In these studies, the digital training group didn’t score significantly better than the traditional group on tooth preparation. Even with the visualization of the three-dimensional images of the tooth preparation, students had difficulty comprehending all the operational flaws within the allowed time [34]. The conventional instructors offered individualized feedback throughout the exercise, providing comprehensive guidance that was proven effective in developing skill proficiency [1]. The aforementioned factors may account for the insignificant difference of all component scores as well as the overall score between the summative (without digital software) and first-session scores. The results equally showed a lack of improvement in students’ autonomous performance when not using digital control softwares. The third-year dental students who participated in this study were in the early stage of preclinical training, so they exhibited a lack of self-assessment skills, which are typically acquired through practical experience in clinical settings [35]. An analytical rubric system provided students with detailed criteria, facilitating a comprehensive understanding of their scores as well as pinpointing their strengths and weaknesses without instructor feedback [3]. Integrating digital scanning and software in the preclinical curriculum fosters valuable evaluation and introduces dental students to their practical application. However, the value of traditional assessment is an irreplaceable element. The role of a teacher extends beyond simply identifying a student’s strength and weakness; it critically involves pointing out the way to correct the flaws or creating a plan to help each student improve upon any deficiencies. While digital software is supplemental for educators to give students more detailed feedback on any defects in preparation, the process of offering constructive criticism requires the thoughtful consideration and nuanced perspective that only a human being can offer [1, 8, 19].

The summative assessment was implemented to assess if students could prepare teeth without digital software. In clinical practice, the assessment of tooth preparations through digital software is convenient and accurate. However, repeated digital scans of crown preparations solely to assess tooth reduction are impractical. The clinician typically assesses the tooth abutment through intraoral scanning after completing all conventional assessments and the operator has deemed the tooth suitable for crown fabrication [36]. Operators are required to demonstrate proficiency in visually assessing preparations, using both the putty index and direct observation. Although students recognize the digital software as a valuable tool within the preclinical laboratory setting, they should develop the capacity for critical analysis and self-evaluation of their work, emulating real-world clinical scenarios and minimizing dependence on digital software [32, 37].

### Student’s perception of digital application in preclinical fixed prosthodontic practice

A comprehensive analysis of the questionnaires revealed a clear trend that the vast majority of students were enthusiastically in favor of incorporating digital systems into their practical training for tooth preparation. An overwhelming majority of students (97.5%) reported the user-friendliness of the digital software, and a significant proportion (92.5 and 85%) showed that digital software aided in improving their preparation and their assessment skills, respectively, in preclinical fixed prosthodontic practice. Various studies also presented a positive outlook regarding handling, pedagogical value, and motivational aspects [14, 27, 38–40]. Students likewise reported that self-assessment using digital software was crucial for visualization and identifying preparation flaws [1, 32]. However, after the summative session without the digital software, 60% of students remained neutral or uncertain regarding self-assessment, this proportion of neutral responses may indicate a need for additional support in fostering students’ confidence in self-assessment. Despite the software’s benefits, students largely disagreed that it could fully replace instructors for guidance (57.5%) or evaluation (42.5%), emphasizing the ongoing necessity of instructors in dental education. Students expressed positive opinions regarding the use of digital software as a supplementary assessment method for tooth preparation procedures because it is valuable for examining different characteristics of teeth, such as damage to adjacent teeth, smoothness of walls, depth of preparation, and convergence/divergence walls [19, 32, 41].

### Limitations

This study’s limitation is the utilization of phantom heads which cannot simulate clinical scenarios, and subsequent investigation is necessary to evaluate and compare the quality of tooth preparations completed by clinical students, and to determine the effectiveness of the digital learning techniques employed. The study performed a comparative analysis of tooth structure reduction according to component and overall surface reduction. The analysis included an average of 24 points (for mesial/distal surface) and 36–48 points (for occlusal/facial/lingual surface). Each point was measured, compared to the rubric, calculated a score, and then averaged for the component/overall reduction. The existing method for comparing points permits the analysis of several points at each surface, as done in some studies [23, 42]. In the future, area-based analysis will be useful for analyzing tooth surface reduction. Increased student participation and implementation of a range of analytical software for evaluating tooth preparations are also recommended. Additional research is necessary to explore the combined implementation of digital and conventional systems. These approaches may allow for a more thorough analysis of dental students’ educational outcomes.

## Conclusion

This study analyzed the efficacy of the digital educational system in students’ crown preparation performance, considering training modalities that included digital softwares but no instructor support. Our conclusions, based on this study’s parameters, are as follows:

1. The pedagogical software improved students’ overall performance in preclinical prosthodontics and facilitated precision in some specific areas of tooth preparation. However, without digital aid, students couldn’t demonstrate their proficiency in tooth preparation autonomously. Although digital softwares facilitate objective feedback on preparation defects, constructive recommendations of the instructor are vital for improving their students’ performance.
2. Students expressed a positive reception to the digital training system in preclinical fixed prosthodontics practice, evidencing its effectiveness for dental undergraduates. It offers students a self-learning opportunity to improve their tooth preparation performance, thus enabling them to refine their skills and techniques.

## Data Availability

The datasets generated and/or analyzed during the current study are available from the corresponding author upon reasonable request.
